# Enhanced immunity in a mouse model of malignant glioma is mediated by a therapeutic ketogenic diet

**DOI:** 10.1186/s12885-016-2337-7

**Published:** 2016-05-13

**Authors:** Danielle M. Lussier, Eric C. Woolf, John L. Johnson, Kenneth S. Brooks, Joseph N. Blattman, Adrienne C. Scheck

**Affiliations:** School of Life Sciences, Arizona State University, Tempe, AZ 85281 USA; Center for Infectious Diseases and Vaccinology, Biodesign Institute, Arizona State University, Tempe, AZ 85281 USA; Neuro-Oncology Research, Barrow Brain Tumor Research Center, Barrow Neurological Institute, St. Joseph’s Hospital and Medical Center, 350 W. Thomas Road, Phoenix, AZ 85013 USA

**Keywords:** Glioblastoma, Glioma, Ketogenic diet, Metabolism, Immunosuppression, Microenvironment, Immune inhibitory checkpoints, Immunology, CTLA-4, PD-1

## Abstract

**Background:**

Glioblastoma multiforme is a highly aggressive brain tumor with a poor prognosis, and advances in treatment have led to only marginal increases in overall survival. We and others have shown previously that the therapeutic ketogenic diet (KD) prolongs survival in mouse models of glioma, explained by both direct tumor growth inhibition and suppression of pro-inflammatory microenvironment conditions. The aim of this study is to assess the effects of the KD on the glioma reactive immune response.

**Methods:**

The GL261-Luc2 intracranial mouse model of glioma was used to investigate the effects of the KD on the tumor-specific immune response. Tumor-infiltrating CD8+ T cells, CD4+ T cells and natural killer (NK) cells were analyzed by flow cytometry. The expression of immune inhibitory receptors cytotoxic T-lymphocyte-associated protein 4 (CTLA-4) and programmed death 1 (PD-1) on CD8+ T cells were also analyzed by flow cytometry. Analysis of intracellular cytokine production was used to determine production of IFN, IL-2 and IFN- in tumor-infiltrating CD8+ T and natural killer (NK) cells and IL-10 production by T regulatory cells.

**Results:**

We demonstrate that mice fed the KD had increased tumor-reactive innate and adaptive immune responses, including increased cytokine production and cytolysis via tumor-reactive CD8+ T cells. Additionally, we saw that mice maintained on the KD had increased CD4 infiltration, while T regulatory cell numbers stayed consistent. Lastly, mice fed the KD had a significant reduction in immune inhibitory receptor expression as well as decreased inhibitory ligand expression on glioma cells.

**Conclusions:**

The KD may work in part as an immune adjuvant, boosting tumor-reactive immune responses in the microenvironment by alleviating immune suppression. This evidence suggests that the KD increases tumor-reactive immune responses, and may have implications in combinational treatment approaches.

**Electronic supplementary material:**

The online version of this article (doi:10.1186/s12885-016-2337-7) contains supplementary material, which is available to authorized users.

## Background

Glioblastoma multiforme (GBM) is a highly aggressive, heterogeneous brain tumor with poor prognosis [1]. Standard of care includes surgical resection followed by radiation and chemotherapy, however median survival is about 15 months with a two-year survival of 30 % and a 5-year survival of <5 % in adults [2]. Despite breakthroughs in our understanding of the disease, therapeutic options available for GBM have remained largely unchanged over the past three decades. This has led to only marginal increases in overall patient survival and new therapeutic approaches to enhance brain tumor treatment are warranted.

One novel therapeutic approach for GBM involves targeting a phenotypic trait shared by virtually all cancer cells, deregulated metabolism. It has been postulated that metabolic alteration such as that seen with the therapeutic ketogenic diet (KD) may be an effective anti-cancer strategy [3]. The KD is a high fat, low-carbohydrate/adequate protein nutritional therapy used in the treatment of refractory epilepsy [4]. We and others have shown that the KD enhances survival in mouse models of malignant glioma [5–8]. We also demonstrated that the KD greatly enhanced survival when administered in combination with radiation [6]. Mechanistically, the KD alters a variety of processes that influence the tumor microenvironment including hypoxia, inflammation, angiogenesis and vascular permeability [5, 9]. However, the effect of a KD on the GBM tumor-reactive immune response has yet to be examined.

We have recently shown that an unrestricted KD decreases expression of the hypoxia marker carbonic anhydrase IX (CAIX) and the key mediator of the hypoxic response hypoxia-inducible factor alpha (HIF-1α) in a mouse model of malignant glioma [9]. Wei et al. demonstrated that hypoxia leads to inhibition of T cell proliferation and effector responses, with induction of CD4 + FoxP3+ T regulatory cells in GBM [10]. This study also demonstrated that this immunosuppressive effect could be reversed by inhibiting HIF-1α. As tumor hypoxia is linked to the less favorable Th2 immune response [11], it is possible that by altering the hypoxic response the KD may promote a Th1 type tumor-reactive immune response. Additionally, we previously demonstrated that the KD reduces activation of the pro-inflammatory transcription factor, nuclear factor kappa B (NF-κB) and reduces expression of cyclooxygenase-2 (COX-2) [5, 9], both of which have been implicated in hypoxia-driven immunosuppression [12]. Taken together these studies led us to hypothesize that the KD may alter the tumor microenvironment to alleviate immune suppression and enhance anti-tumor immunity.

In this paper we investigated the role that an unrestricted KD plays in alleviating tumor immune suppression in a mouse model of malignant glioma. We studied the direct effects of this metabolic therapy on total infiltration and function of tumor-reactive T cells and natural killer (NK) cells, as well as the indirect benefits of this metabolic therapy on alleviation of immune suppression in the tumor microenvironment.

## Methods

### Antibodies and cell lines

Fluorochrome-conjugated anti-mouse monoclonal antibodies (Abs) specific for CD8α, CD274, CD279, CTLA-4, CD86, tumor necrosis factor (TNF), interferon gamma (IFNγ), interleukin-2 (IL-2), CD4, FoxP3, NKp46, CD3, and interleukin-10 (IL-10) were purchased from eBiosciences (San Diego, CA) and diluted 1:200 prior to use. Anti-CD8 depletion antibodies were purified from the mouse 2.43 hybridoma cell line purchased from ATCC (Manassas, VA). Bioluminescent GL261-Luc 2 cells were derived and grown as previously described [6].

### Mice and tumor implantation

GL261-Luc2 cells were harvested by trypsinization, washed and resuspended at a concentration of 1–2x10^7^ cells/ml in DMEM without FCS and implanted into ten week old B6 (Cg)-*Tyr*^*c-23*^/J (albino C57BL/6) mice (The Jackson Laboratory, Bar Harbor, ME) at an average weight of 19–20 g as previously described [5, 6, 13]. Briefly, animals were anesthetized by an intraperitoneal injection of ketamine (10 mg/kg) and xylazine (80 mg/kg), placed in a stereotactic apparatus and an incision was made over the cranial midline. A burrhole was made 0.1 mm posterior to the bregma and 2.3 mm to the right of the midline. A needle was inserted to a depth of 3 mm and withdrawn 0.4 mm to a depth of 2.6 mm. Two μl of GL261-luc2 cells (10^7^ cells/ml) were infused over the course of 3 min. The burrhole was closed with bonewax and the incision was sutured.

### Treatment and animal monitoring

Following implantation surgery, animals were fed standard rodent chow for 3 days. Animals were then randomized to remain on standard diet (SD) or changed to a KD (KetoCal®; Nutricia North America, Gaithersburg, MD). The KD was obtained directly from the manufacturer and is a nutritionally complete diet providing a 4:1 ratio of fats to carbohydrates plus protein (72 % fat, 15 % protein, and 3 % carbohydrate). The KD was prepared by mixing KetoCal® with water (2:1) and fed to the animals each day (*ad libitum*). Bioluminescence was analyzed to quantify tumor burden as described [6]. Serum β-hydroxybutyrate (βHB) and glucose levels were measured using a Precision Xtra® blood monitoring system (Abbott Laboratories, Abbott Park, IL). Animals were weighed every 3–5 days and euthanized upon occurrence of visible symptoms of impending death such as hunched posture, reduced mobility and weight loss [5, 14]. Measurements of animal body weight, blood βHB, and glucose can be found in (Additional file 1: Figure S1).

### CD8 depletion *in vivo*

Supernatant from 2.43 hybridoma cells was precipitated in saturated ammonium sulfate to 45 % (v/v) overnight at 4 °C and dialyzed against PBS for 24 h. The concentration of dialyzed antibody was determined by UV spectroscopy, and 0.3 mg of purified antibody was administered via intraperitoneal injection twice before tumor inoculation (day −5 and −3), and continued every three days after inoculation until euthanasia. CD8 T cell depletion was confirmed by flow cytometry analysis of peripheral blood mononuclear cells, as previously described [15]. Confirmation of CD8 depletion can be found in (Additional file 2: Figure S2).

### Tissue preparation

When mice became symptomatic they were anesthetized with 80 mg/kg ketamine, 10 mg/kg xylazine followed by cardiac perfusion with ice-cold RPMI media just prior to euthanization. Tumor tissue and non-tumor contralateral brain were collected in RPMI media and run through a 70 μm filter. Tumor-infiltrating cells were isolated from tumor tissue by centrifugation over a 30/70 % Percoll gradient (Sigma-Aldrich, St. Louis, MO) before antibody staining and analysis of cell populations on an LSRFortessa flow cytometer (BD Biosciences, San Jose, CA). Flow cytometry data were analyzed with FlowJo8.8 (Tree Star Inc., Ashland, OR) and graphs were generated using Prism 5 software (GraphPad Software, La Jolla, CA). Gating strategies and isotype controls can be found in the Additional file 3: Figure S3 and Additional file 4: Figure S4 section.

### Intracellular cytokine staining

Lymphocytes were cultured alone or stimulated with GL261-Luc2 cells at a density of 10^6^ cells per well (6-well plate). GolgiStop (BD Biosciences) was added at 1 h to inhibit export of cytokines and after a further 5 h of incubation, cells were stained for extracellular proteins. Permeabilization and intracellular staining for cytokines was done according to manufacturer’s instructions using the Cytofix/Cytoperm kit (BD Biosciences). Gating strategies and isotype controls can be found in the Additional file 5: Figure S5 and Additional file 6: Figure S6 section.

### Cytotoxicity ELISA

Lymphocytes were isolated from tumor tissue, and cultured alone or with GL261-Luc2 cells at varying effector to target cell ratios. Lactate dehydrogenase (LDH) ELISA was performed using CytoTox 96 Non-Radioactive Cytotoxicity Assay (Promega, Madison, WI). Absorbance was recorded at 490 nm.

### Animals and virus

Six to 8-week-old female C57BL/6 mice were obtained from The Jackson Laboratory. All experiments were conducted under Arizona State University IACUC approval and followed all relevant federal guidelines and institutional policies. The Armstrong and clone 13 strains of Lymphocytic Choriomeningitis Virus (LCMV) were grown as previously described [16]. Mice were infected with 2 x 10^5^ PFU of LCMV (Armstrong) injected intraperitoneally or 2 x 10^6^ PFU of LCMV (clone 13) injected intravenously.

### Statistical methods

Statistical analyses were performed using GraphPad Prism 5 (GraphPad Software, San Diego, CA). All values are represented as the mean ± SD and significance was determined using both the Student’s *t* test and the Mann Whitney non-parametric test. *P* < 0.05 was considered statistically significant. For the Kaplan Meier survival data the log-rank (Mantel-Cox) test was used to assess statistical significance.

## Results

### KD enhanced survival is mediated by CD8+ T cells

**Fig. 1:**
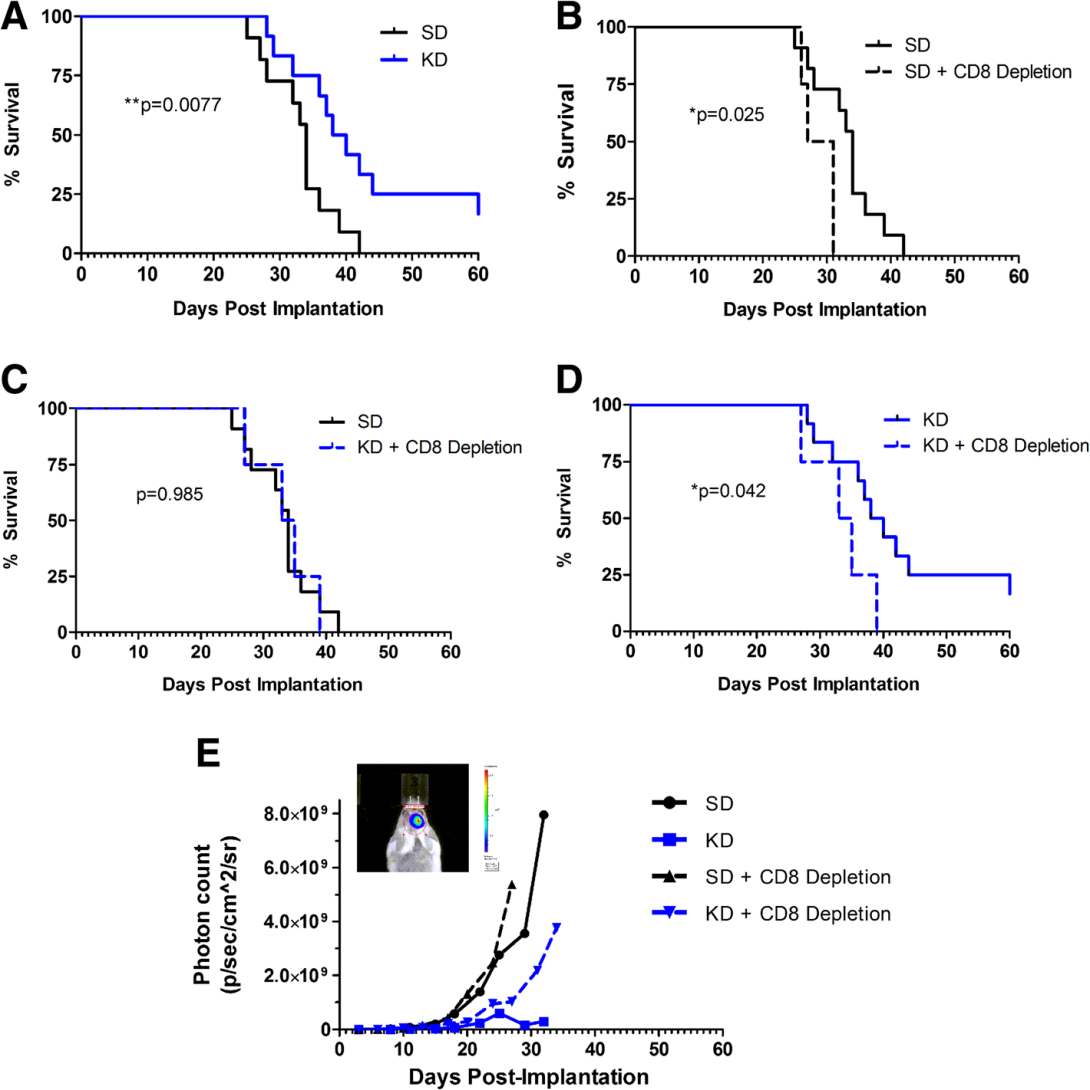
Enhanced survival with the ketogenic diet is mediated in part by CD8 T cells. Kaplan-Meier survival curves for ketogenic diet (KD) versus standard diet (SD) (**a**), SD versus SD + CD8 depletion (**b**), SD versus KD + CD8 depletion (**c**), KD versus KD + CD8 depletion (**d**). Bioluminescent tumor signals plotted as *in vivo* photon count versus days post-implantation (**e**). *N* = 12 for immune competent mice; *N* = 5 for CD8 depleted mice; Log-rank (Mantel-Cox) test; p-values indicated on graphs

Tumor bearing animals maintained on the ketogenic diet (KD) had a greater median survival when compared to animals fed a standard diet (SD) (Fig. 1a). In order to effectively evaluate the importance of tumor-reactive CD8+ T cells in slowing tumor progression, CD8+ T cells were depleted from immune competent albino C57BL/6 mice bearing tumors. There was a significant decrease in survival of mice depleted of CD8+ T cells prior to tumor cell inoculation in comparison to wild type mice maintained on SD (Fig. 1b). In order to determine the importance of CD8+ T cells in the anti-tumor effects of the KD, CD8+ T cell depleted animals were treated with the KD and survival was measured. Although the KD significantly improved survival in immune intact mice when compared to those maintained on SD, that difference in survival is lost when CD8+ T cells are depleted and mice are treated with the KD in comparison to immune intact mice fed SD (Fig. 1c). Furthermore, the KD significantly increased survival in immune intact mice when compared to CD8 depleted mice fed KD (Fig. 1d). Analysis of bioluminescence data also shows slower tumor growth in animals treated with KD when compared to SD in both immune competent and CD8 depleted mice (Fig. 1e).

### The KD enhances immune cell infiltration, and increases the ratio of tumor-reactive CD4+ T cells to Treg ratio

**Fig. 2:**
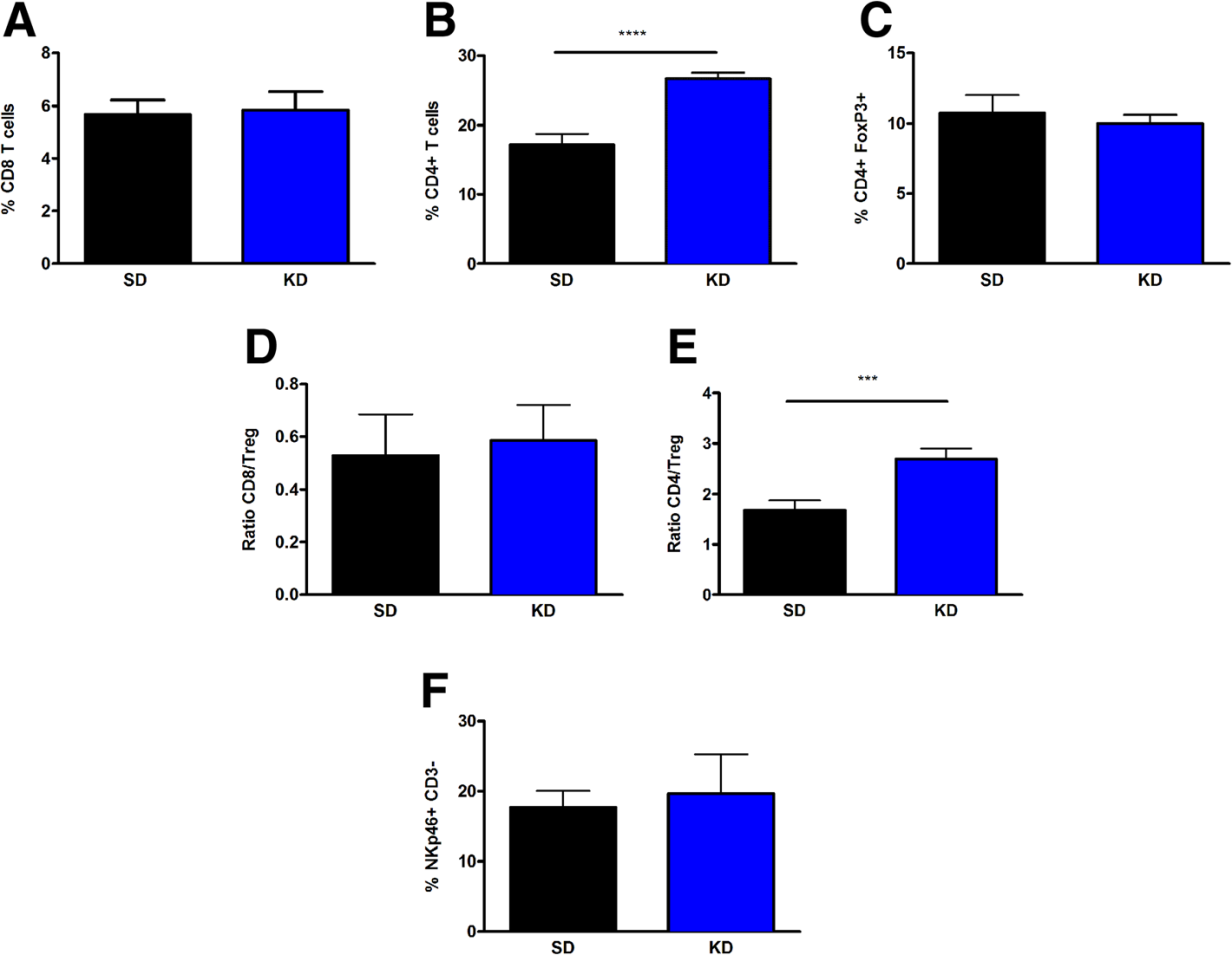
CD4+ T cell infiltration increases in mice fed the KD, without increases in Treg cell numbers. Flow cytometry analysis was performed to assess the cell types infiltrating tumors from mice fed both SD and KD. CD8 T cells (**a**), CD4 T cells (**b**) and CD4 + FoxP3+ T regulatory cells (**c**) were assessed. The ratio of CD8 T cells to T regulatory cells (**d**) and CD4 to T regulatory cells (**e**) were determined. The percent of infiltrating NKp46 + CD3- natural killer cells (**f**) were also assessed. *N* = 5; student’s two-tailed *t*-test; ***p < 0.001; ****p < 0.0001

To evaluate the effects of the KD on immune cell infiltration into the tumor, amounts of tumor-infiltrating CD8+, CD4+, CD4 + FoxP3+, and NKp46 + CD3- cells were tested. There was no significant difference in the percentage of tumor-infiltrating CD8+ T cells between mice fed the KD and the SD (Fig. 2a). However, mice fed the KD had a significant increase in the percentage of CD4+ T cells infiltrating the tumor in comparison to SD (Fig. 2b). This increase in the percentage of CD4+ T cells was not due to an increase in the percentage of FoxP3 + CD4+ T regulatory (Treg) cells (Fig. 2c), and therefore the ratio of CD4+ T cells to Treg cells is significantly increased in tumors from mice fed a KD (Fig. 2e). In comparison, the CD8+ T cell to Treg cell ratio remained unchanged when comparing the two treatment groups (Fig. 2d). Lastly, there was no difference in the percentage of tumor-infiltrating NK cells in tumors from mice fed a KD compared to SD (Fig. 2f). Similar results were found when looking at the total number of infiltrating cells (data not shown). Therefore, the KD enhances CD4+ T cell presence at the tumor site, and this increase is not associated with an increase in the T regulatory cell subset.

### The KD influences expression of immune inhibitory receptors on tumor-infiltrating lymphocytes, and immune inhibitory ligands on glioma cells

**Fig. 3:**
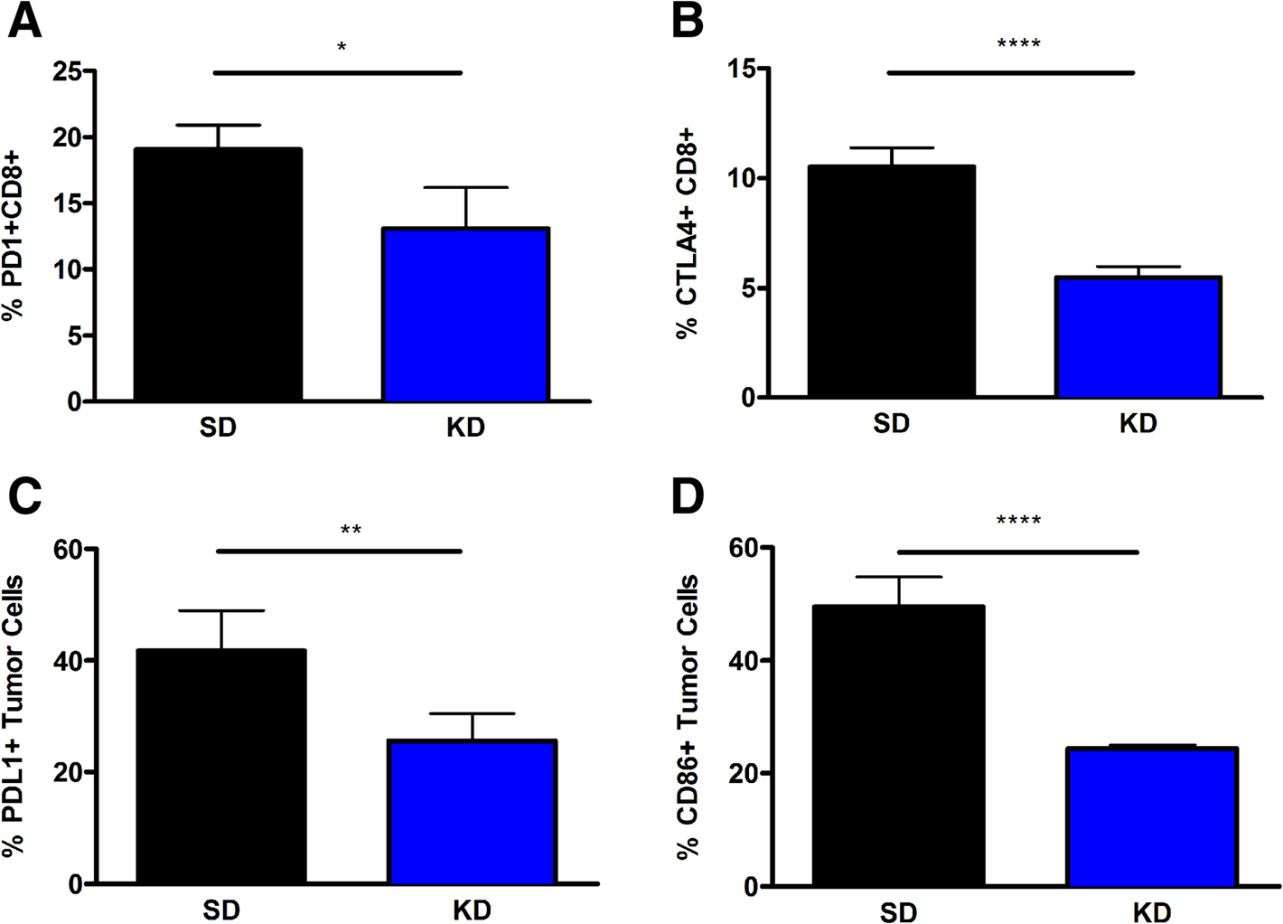
The ketogenic diet reduces expression of immune inhibitory receptors and ligands expressed in glioma tumors. Expression of the immune inhibitory receptors, PD-1 (**a**) and CTLA-4 (**b**) on infiltrating CD8 T cells isolated from tumors from mice fed each diet were assessed. Expression of the immune inhibitory ligands, CD86 (**c**) and PD-L1 (**d**), on GL261-Luc2 tumor tissue was also assessed. *N* = 5; student’s two tailed *t*-test; *p < 0.05; **p < 0.01; ****p < 0.0001

Tumor cell expression of immune inhibitory checkpoint proteins is a major mechanism by which tumors limit the efficacy of immune responses *in vivo*. To examine the influence of the KD on immune inhibitory checkpoints we evaluated changes of immune inhibitory receptor expression on CD8+ tumor-infiltrating lymphocytes (TILs), and changes in expression of inhibitory ligands on the tumor cells. Mice fed the KD had significantly reduced expression of two inhibitory ligands, PD-1 (Fig. 3a) and CTLA-4 on CD8+ TILs (Fig. 3b). Additionally, mice fed the KD had reduced expression of CD86 (Fig. 3c) and PD-L1 (Fig. 3d) on the tumor cells. This suggests that the KD may alter tumor-mediated T cell suppression by reducing the number of cells that are susceptible to inhibition through the PD-1 and CTLA-4 inhibitory pathways.

### The KD enhances innate and adaptive tumor specific immune function against glioma cells

**Fig. 4:**
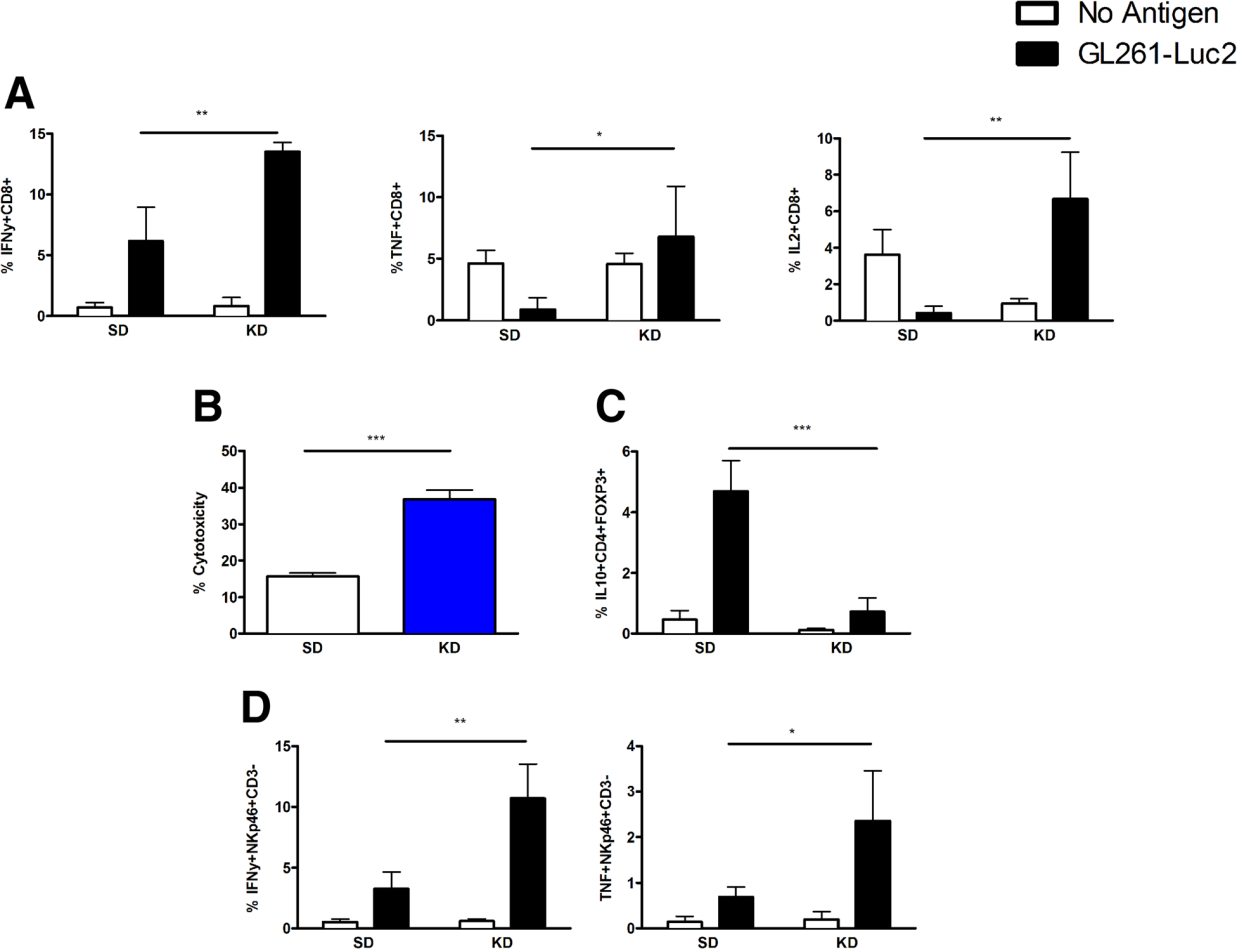
The ketogenic diet significantly enhances tumor-reactive CD8+ T cell and NK cell activity. Tumor-infiltrating lymphocytes (TILs) isolated from gliomas from mice fed KD versus SD were cultured alone (white bar) or in the presence of GL261-Luc2 tumor cells (black bar) to access activity. Analysis of IFNγ, TNF and IL-2 production in tumor-infiltrating CD8+ T cells was performed (**a**). Cytotoxic capability of CD8+ T cells isolated from tumors was assessed following exposure to GL261-Luc2 cells (**b**). IL-10-production in CD4 + FoxP3+ T regulatory cells was also assessed in response to stimulation with GL261-Luc2 cells (**c**). IFNγ and TNF production in the infiltrating NKp46 + CD3- natural killer cells isolated from tumors were assessed (**d**). *N* = 5; student’s two-tailed *t*-test between the antigen-challenged SD and KD groups only; *p < 0.05; **p < 0.01; ***p < 0.001

To evaluate the influence of the KD on tumor-reactive immune cells at the tumor site, immune cell function from TILs removed from the tumor site at time of necropsy was tested. Intracellular cytokine staining following stimulation with tumor cells showed that when compared to SD, the KD significantly increases the ability of tumor-reactive CD8+ T cells to produce interferon gamma (IFNγ), tumor necrosis factor (TNF), and interleukin 2 (IL-2) when stimulated with GL261-Luc2 cells (Fig. 4a). Additionally, the KD significantly increased cytotoxic capabilities of tumor-reactive T cells from mice when compared to SD (Fig. 4b). The function of T regulatory cells was also assessed by intracellular cytokine staining for interleukin 10 (IL-10). Although we did not find a difference in the number of tumor-infiltrating Tregs, those found in the tumors from animals fed the KD produced significantly less IL-10 in response to GL261-Luc2 cells when compared to animals maintained on SD (Fig. 4c). Lastly, we studied natural killer (NK) cell function and found that tumor-infiltrating NK cells from mice fed the KD produce significantly more IFNγ and TNF in response to GL261-Luc2 cells than the cells isolated from SD fed animals (Fig. 4d). Whether through direct interaction with immune cells, or through alleviation of tumor immune suppression in the microenvironment, the KD significantly enhances tumor-reactive immune function.

### The KD enhances innate and adaptive tumor-reactive immune responses indirectly via alleviation of immune suppression

**Fig. 5:**
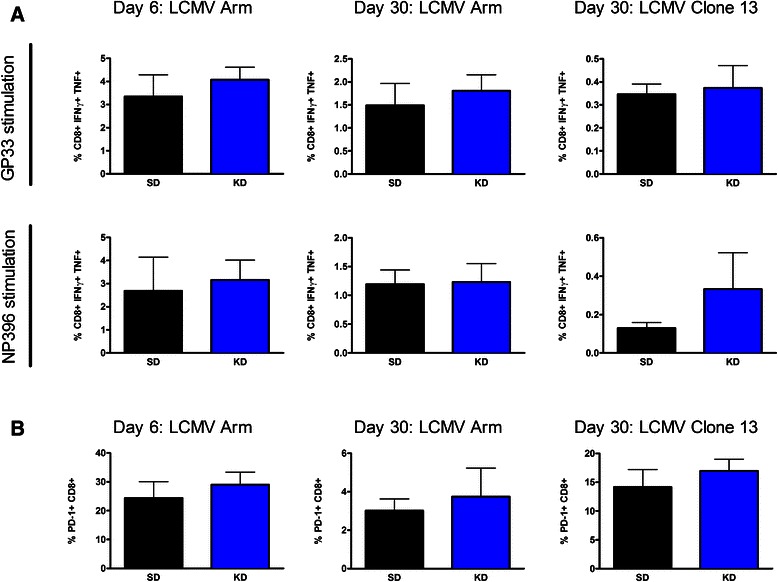
The ketogenic diet had no effect on T cell activity in an acute and chronic mouse model of LCMV infection. Splenocytes from non-tumor bearing mice infected with LCMV Armstrong or Clone 13 were isolated at day 6 and 30, and stimulated with GP33 or NP396 antigens. IFNγ + TNF + CD8+ cells in mice fed SD versus KD (**a**). PD-1 + CD8+ expression in mice fed SD versus KD (**b**). *N* = 5 in each group

To determine if the KD specifically enhances TIL function in the tumor microenvironment or alters global immune status, the effects of the KD on immune responses to two strains of Lymphocytic Choriomeningitis Virus (LCMV) was examined. Non-tumor bearing mice were infected with either LCMV Armstrong or Clone 13, and CD8 T cell function was accessed at Day 6 and 30. There was no significant difference in cytokine production by CD8+ T cells responding to either LCMV dominant epitopes, GP33 or NP396 (Fig. 5a) at either time point or with either infection regardless of diet. Additionally, there was no significant difference in the percentage of PD-1 + CD8+ T cells between KD and SD fed mice (Fig. 5b). Although the KD did not alter CD8 function against acute and chronic viral infections, it did alter immune mediated killing at the tumor site suggesting alleviation of immune suppression is specific to the tumor microenvironment.

## Discussion

Activated effector immune responses against glioblastoma multiforme (GBM) may provide benefits in patient survival; however these tumors exert a variety of immunosuppressive pressures on the surrounding microenvironment [17, 18]. These include increased induction of CD8 + FOXp3+ regulatory T cells (Tregs), elevated immunosuppressive cytokine levels, diminished CD4+ helper T cell populations, tolerized antigen presenting cells and upregulated immune inhibitory checkpoints [19]. For example, Tregs suppress immune responses by secreting cytokines such as IL-10 and facilitating inactivation of CD8+ cytotoxic T cells by direct cell-to-cell interactions [20]. A key observation in immunosuppressed GBM patients is a decrease in CD4+ T cells with an increased proportion of Tregs and increased IL-10 levels [19, 21]. The current study demonstrated that tumors from animals maintained on the KD had a significantly increased CD4+ T cell population and a decreased proportion of Tregs when compared to control animals. Further the Tregs isolated from animals maintained on the KD produced significantly less IL-10 when stimulated with tumor cells. Similar results were demonstrated in a study using a pancreatic cancer model which showed increased CD4+ T cells and decreased Tregs when animals were fed a KD [22].

In addition to increasing Tregs and IL-10 production in the microenvironment, tumors exploit immune inhibitory signaling pathways involving direct cell-to-cell interactions. Key mediators of this system include cytotoxic T-lymphocyte-associated protein 4 (CTLA-4) and programmed death-1 (PD-1) which are found on the surface of activated effector T cells and act as checkpoints to regulate immune proliferation and activation. For example, when PD-1 binds its ligand, programmed death ligand 1 (PD-L1), activated CD8+ and CD4+ T cells are suppressed [23]. Increased PD-L1 expression has been observed on tumor cells and immune cells within the GBM microenvironment [24–27] and leads to direct inactivation of CD8+ T cells [28, 29]. The current study demonstrates significantly decreased expression of PD-1 and CTLA-4 on tumor-infiltrating CD8+ T cells and decreased expression of their ligands (PD-L1 and CD86, respectively) on dissociated tumor cells from animals maintained on the KD when compared to control animals. Blockade of the CTLA-4 and PD1 immune checkpoints represents a potentially important anti-glioma strategy that has proven effective in preclinical models of glioma [30–34] and has warranted exploration in ongoing clinical trials [35].

The current study suggests that the KD may shift the immunological landscape from inflammatory, non-protective immune responses to cytotoxic Th1 responses and promotion of immune mediated killing at the tumor site. Shifting the balance toward a Th1 immune response leads to a general change in cytokine milieu at the tumor site which alters antigen presenting cell maturation and amount of overall immune cell activation [36–41]. This may explain results seen in this manuscript including increased NK and CD8+ T cell function, changes in CD4+ T cell recruitment, reduction in immune inhibitory receptor expression, and ligand availability on the tumor cells themselves. It should be noted that increased CD4 to CD8 T cell ratio may be indicative of a Th2 type immune response at the tumor site [42], which may promote an immune tolerance state; however, greater CD8 T cell activation in the tumors from mice maintained on a KD suggests this is not the case.

It is known that activated T cells undergo metabolic reprogramming in which glycolysis is required to support proliferation and efficient growth [43–46]. Recent evidence also suggests that reduced glucose availability and increased fatty acid oxidation favors T regulatory cells over effector T cells [47]. However, tumor-infiltrating T cells from mice fed the KD are still able to mount effective responses, undergo appropriate differentiation, and retain function even with the characteristic drop in glucose availability that accompanies the KD. It is currently unclear how the KD alters the metabolic activity of lymphocytes and why this effect appears to be specific to the lymphocytes isolated from the tumor microenvironment. It is possible that T-cells can utilize ketones as a primary energy source in place of glucose in a way similar to that of normal cells in the brain [48, 49]. Recent work has suggested that tumor cells may outcompete other cells in the microenvironment for glucose and other nutrients, thereby reducing the activation of anti-tumor effector T cells [50, 51]. By providing ketones as an alternative energy source for lymphocytes it can be postulated that the KD may alleviate immunosuppression mediated by nutrient competition. Further studies are needed to explore this question and determine the precise role of ketones in T cell metabolism.

While the effect of the KD on tumor-infiltrating lymphocytes has only recently been explored, existing preclinical *in vitro* and *in vivo* data as well as case reports and anecdotal information have generated increased support for clinical testing. Prospective Phase I and II clinical trials have been initiated to assess the safety, efficacy and tolerability of the KD in patients with recurrent GBM (ClinicalTrials.gov; NCT01754350; NCT01535911; NCT01865162; NCT02149459). In addition, we have initiated a phase I/II trial assessing the tolerability and efficacy of the KD up-front, concurrently with radiation and temozolomide in newly diagnosed GBM patients (NCT02046187) based on our preclinical data demonstrating that the KD, when given in combination with radiation, dramatically enhances survival when compared to radiation treatment alone [6]. The mechanisms underlying this effect are still under investigation; however, as radiation-induced tumor killing is known to expose the immune system to a greater diversity of tumor antigens, increased antigen processing, and increased immunogenic cytotoxicity it is possible that the KD as an adjuvant can work to augment the effect of radiation in part by enhancing immunity against GBM.

## Conclusions

In summary, the KD may work as an immune adjuvant in the glioma microenvironment by reducing immune suppression, and promoting Th1 type immune responses against the tumor. These data provide additional support for the use of the KD in combination with the current standard of care and newer therapies for the treatment of brain tumors.

### Ethics statement

This study was performed in strict accordance with the recommendations in the Guide for the Care and Use of Laboratory Animals of the National Institutes of Health. The protocol was approved by the Institutional Animal Care and Use Committee of St. Joseph’s Hospital and Medical Center (protocol number 334 (A3510-01)). All surgery was performed under ketamine/xylazine anesthesia, and every effort was made to minimize suffering.

### Consent for publication

Not applicable.

### Availability of data and materials

The datasets supporting the conclusions of this article are included within the article and its supplementary files.

## Additional files

Additional file 1: Figure S1. βHB, glucose and weight measurements. Blood ketone and glucose measurements taken at days 7 and 14 post-implantation show (A) higher βHB and (B) lower glucose in KD treated animals (C) weight measurements were taken every 3–5 days. Graph shows weights normalized to the average starting weight of each group. *N* = 12 for immune competent mice; *N* = 5 for CD8 depleted mice; student’s two-tailed *t*-test; **p* < 0.05; ***p* < 0.01; ****p* < 0.001; *****p* < 0.0001. (TIF 24634 kb) Additional file 2: Figure S2. Confirmation of CD8 depletion in 2.43 treated mice. PBMCs were collected on day 7 post tumor inoculation during CD8 depletion. PBMCs stained from mice fed KD versus SD, analyzed using Flowjo 8.8.7. Percentage of CD8+ T cells in blood indicated on plot. (TIF 3065 kb) Additional file 3: Figure S3. Gating strategies for CD8+, CD4+, and Treg T cells. Flow cytometry analysis was performed to assess the cell types infiltrating tumors from mice fed both SD and KD. Percentage of CD8 T cells (A), CD4 T cells (B) and CD4 + FoxP3+ T regulatory cells (C) were assessed. (TIF 8002 kb) Additional file 4: Figure S4. Gating strategies for inhibitory receptor expression on CD8+ T cells. Flow cytometry analysis was performed to assess the inhibitory receptor expression on tumor-infiltrating CD8+ T cells from mice fed both SD and KD. Percentages of PD-1+ (A) and CTLA-4+ (B) are indicated on flow plots. Data gating on CD8+ T cells. (TIF 6955 kb) Additional file 5: Figure S5. Gating strategies for cytokine expression of CD8+ T cells. Tumor-infiltrating lymphocytes (TILs) isolated from gliomas from mice fed KD versus SD were cultured alone or in the presence of GL261-Luc2 tumor cells. Analysis of IFNγ (A), TNF (B) and IL-2 (C) production in tumor-infiltrating CD8+ T cells was performed. Percentage of positive cells indicated on plots. (TIF 9343 kb) Additional file 6: Figure S6. Gating strategies for cytokine expression of T regulatory cells and NK cells. Tumor-infiltrating lymphocytes isolated from gliomas from mice fed KD versus SD were cultured alone or in the presence of GL261-Luc2 tumor cells. Analysis of IL-10 + Treg cells (A), IFNγ + NK cells (B), and TNF + NK cells (C) was performed. Percentage of positive cells indicated on plots. (TIF 10675 kb)

## Abbreviations

GBM: glioblastoma multiforme
KD: ketogenic diet
SD: standard rodent diet
TIL: tumor-infiltrating lymphocyte
CTLA-4: cytotoxic T-lymphocyte-associated protein 4
PD-1: programmed death 1
βHB: β-hydroxybutyrate
TNF: tumor necrosis factor
IFNγ: interferon gamma
IL: interleukin
